# Atlanta Medical College

**Published:** 1876-07

**Authors:** 


Dr. Powell—*Sir:* Is it true that there is a department of pharmacy in the Atlanta Medical College. * * * How many did they graduate at their last Commencement? * * * Have they any apparatus for the illustration of practical teaching of pharmaceutical knowledge? Can the faculty of any regular Medical College, through the chairs of Materia Medica and Chemistry, teach the manipulations and impart the minute details of knowledge necessary to fit the student for the responsible and important duties of a practical pharmaceutist? Indeed, would it be possible for even an experienced and learned professor of pharmacy to do it with the limited time and facilities possessed by these Institutions? I do not make these inquiries to reflect upon the Atlanta College, but from a sense of duty to the profession and in view of the great importance of the subject. I hope you will answer them fully, that we may judge for ourselves.

The above letter, from one of our most esteemed friends, has, for some time, been on hand, and the answer delayed in order to obtain the requisite information for reply. It is true that the Atlanta Medical College has a Chair of Pharmacy, "so-called," authorized by the Legislature about two years ago; but, we are informed, have had no special, separate, regularly elected professors in charge of this department. Nor have they had any proper outfit of apparatus or material for the teaching of this very responsible and important branch of study. Yet, at the close of the last term, they turned out upon the world eighteen graduates in pharmacy, nearly all of whom, we are reliably informed, had attended but a single short session of lectures. That "the Faculty of any regular medical college, through the chairs of materia medica and chemistry," as usually conducted, could "teach the manipulations and impart the minute details of knowledge necessary to fit the student for the responsible and important duties of a practical pharmaceutist," is, in our judgment, too manifestly impossible to demand an argument at our hands. Nor would it be possible in so short a period, and with such limited facilities, for "the most learned and experienced" teacher to fit a student for the practical duties in this intricate and important science. We will venture the assertion that there are very few graduates of our best medical colleges who are prepared at once to discharge the duties of a clerk in a drug store, much less to prepare all the therapeutical preparations and chemicals from the crude materials. This is our opinion, but if the learned professors in the established pharmaceutical colleges and the professors of chemistry and materia medica in our first-class medical colleges will say we are wrong, we will say no more on this subject. We await their answer.

In thus candidly answering the inquiry of our correspondent we are prompted alone by a sense of duty to the profession. As public journalists we are expected to speak the truth, and to advocate correct principles, without fear or favor; and we should be pleased to hear from any of our brethren on this point. Our journal is open for a free expression upon all matters of importance and interest to the profession.

